# Single-Cell, Genome-wide Sequencing Identifies Clonal Somatic Copy-Number Variation in the Human Brain

**DOI:** 10.1016/j.celrep.2014.07.043

**Published:** 2014-08-21

**Authors:** Xuyu Cai, Gilad D. Evrony, Hillel S. Lehmann, Princess C. Elhosary, Bhaven K. Mehta, Annapurna Poduri, Christopher A. Walsh

**Affiliations:** 1 Division of Genetics and Genomics, Manton Center for Orphan Disease Research and Howard Hughes Medical Institute, Boston Children’s Hospital, Boston, MA 02115, USA; 2 Department of Pediatrics, Harvard Medical School, Boston, MA 02115, USA; 3 Program in Medical and Population Genetics, Broad Institute of MIT and Harvard, Cambridge, MA 02138, USA; 4 Program of Biological and Biomedical Sciences, Harvard Medical School, Boston, MA 02115, USA; 5 Department of Neurology, Boston Children’s Hospital and Harvard Medical School, Boston, MA 02115, USA

## Abstract

De novo copy-number variants (CNVs) can cause neuropsychiatric disease, but the degree to which they occur somatically, and during development, is unknown. Single-cell whole-genome sequencing (WGS) in >200 single cells, including >160 neurons from three normal and two pathological human brains, sensitively identified germline trisomy of chromosome 18 but found most (≥95%) neurons in normal brain tissue to be euploid. Analysis of a patient with hemimegalencephaly (HMG) due to a somatic CNV of chromosome 1q found unexpected tetrasomy 1q in ~20% of neurons, suggesting that CNVs in a minority of cells can cause widespread brain dysfunction. Single-cell analysis identified large (>1 Mb) clonal CNVs in lymphoblasts and in single neurons from normal human brain tissue, suggesting that some CNVs occur during neurogenesis. Many neurons contained one or more large candidate private CNVs, including one at chromosome 15q13.2-13.3, a site of duplication in neuropsychiatric conditions. Large private and clonal somatic CNVs occur in normal and diseased human brains.

## INTRODUCTION

Several recent studies have implicated somatic mutations in a range of diseases, including neurodevelopmental disorders (Biesecker and Spinner, 2013; Jamuar et al., 2014; Poduri et al., 2013; Veltman and Brunner, 2012), with the manifestations of somatic disorders determined by the mutation, its prevalence in the tissue, and the time point during development when the mutation occurred (Poduri et al., 2013). Single-nucleotide variants (SNVs) and copy-number variants (CNVs) arising during prenatal brain development have been linked to brain malformations such as hemimegalencephaly (HMG), in which one hemisphere of the brain is abnormally enlarged, resulting in severe neurological defects, including epilepsy and intellectual disability (Lee et al., 2012; Poduri et al., 2012).

Somatic aneuploidy has been proposed as a mechanism to generate normal genetic diversity in neurons (Bushman and Chun, 2013). Previous reports suggested aneuploidy rates of 1.3%–40%, potentially increasing with age (Kingsbury et al., 2005). Advances in single-cell genomics allow direct assessment of single neuronal genomes from postmortem human brains, enabling systematic characterization of somatic aneuploidies and subchromosomal CNVs (Evrony et al., 2012; Gole et al., 2013; McConnell et al., 2013).

Since all current single-cell studies rely on genome amplification, which introduces biases and artifacts, we performed CNV analysis of single human neurons using two different methods: multiple displacement amplification (MDA)-based single-cell whole-genome amplification (scWGA) (Evrony et al., 2012; Wang et al., 2012; Xu et al., 2012) and a PCR-based method known as GenomePlex, marketed by Sigma (Van der Aa et al., 2013; Voet et al., 2013; Yin et al., 2013). Our analysis of single neuronal genomes from normal and diseased human brains with both MDA and GenomePlex shows, consistent with recent reports, that somatic aneuploidy is rare but somatic CNVs are not rare (Gole et al., 2013; McConnell et al., 2013). We also show that clonal somatic CNVs exist both in normal brain and in HMG.

## RESULTS

### scWGA by MDA and GenomePlex

**Figure 1:**
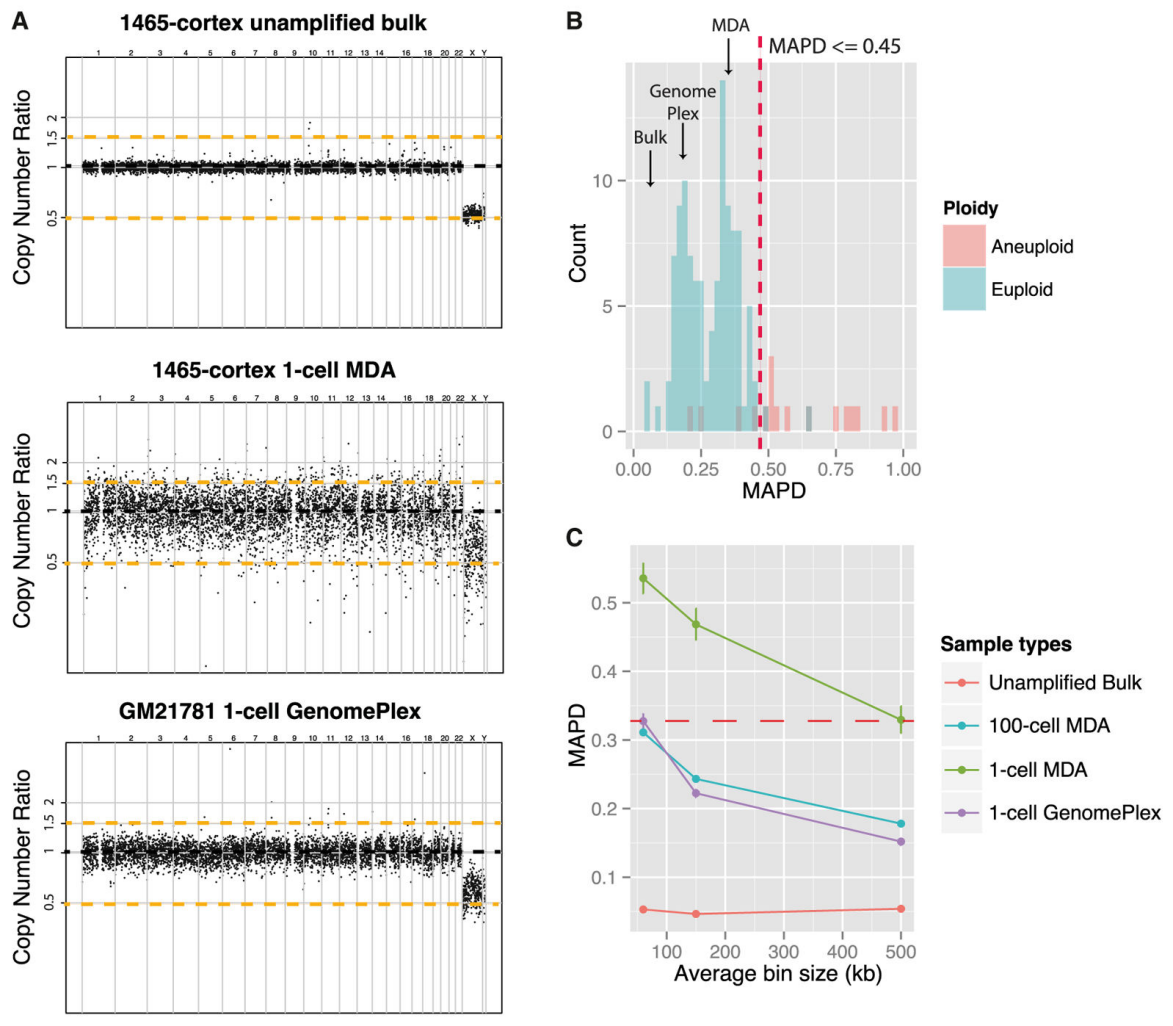
CN Analysis from Low-Coverage WGS (A) Comparison of WGS CN analysis in unamplified DNA, single-cell MDA, and single-cell GenomePlex. The x axis represents 6,000 bins across the genome. The y axis represents the log2 CN ratio of each bin relative to the expected CN based on a simulated reference (see Experimental Procedures). Black dashed lines indicate CN of 2 and orange dashed lines indicate CN of 1 and 3. Each chromosome is divided by vertical lines and labeled on top of the graph. Note that unamplified bulk DNA gives the clearest signal, while single-cell samples are noisier, especially MDA, but still allow recognition of sex chromosome differences in these male samples. (B) MAPD scores for WGS CN analysis of unamplified DNA samples, and single-cell genomes analyzed using GenomePlex or MDA. The histogram shows MAPD scores of all sequenced samples from normal individuals. Bulk (unamplified) DNA gives the best (lowest) MAPD scores (mean = 0.06 ± 0.02, n = 3), whereas GenomePlex (mean = 0.20 ± 0.05, n = 54) gives generally lower MAPD scores than MDA (mean = 0.45 ± 0.17, n = 89), and MDA samples form a long tail of high MAPD scores suggesting low quality (also see Figure S1). Most samples were called euploid (n = 111) and fewer were called aneuploid (n = 17), and most aneuploid cells had high MAPD scores suggesting unreliable calls. A total of 113 samples passed MAPD threshold ≤ 0.45 (red dash line) and 15 samples failed, all amplified by MDA, including three that failed initial multiplex-PCR quality control. Among samples with MAPD ≤ 0.45, 109 out of 113 were euploid and 4 out of 113 were potentially aneuploid. (C) Effect of bin size on CN data noise. Average MAPD score of bulk, MDA, and GenomePlex single-cell samples plotted for 500 kb (6,000 bins total), 150 kb (20,000 bins), and 60 kb (50,000 bins) bins. At each bin size, the average reads per bin is normalized to ~500 for MDA and ~250 for GenomePlex and bulk samples. MAPD of both MDA and GenomePlex samples increases with decreasing average bin size, whereas the MAPD score of bulk DNA remains unchanged with bin sizes, suggesting that both amplifications introduce more prominent noise at smaller local regions (n = 2 for bulk; n = 4 for MDA single cells; n = 1 for MDA 100-cell; and n = 4 for GenomePlex single cells; error bar, ±SD). The red dashed line indicates that the CN noise of MDA samples at 500 kb bin size is comparable to the CN noise of GenomePlex single-cell samples at 60 kb average bin size; MAPD scores equal 0.33 ± 0.02 and 0.33 ± 0.01 (error = ±SD), respectively. The increased noise from MDA samples can be partially compensated by reducing CN resolution. GenomePlex data are from Navin et al. (2011), due to insufficient sequencing depth of our own GenomePlex data, and were used to compare with four wild-type single-cortical neurons and one 100-caudate neuron sample amplified by MDA.

We isolated single neuronal nuclei by fluorescence-activated cell sorting (FACS) as previously described (Evrony et al., 2012), using NeuN immunoreactivity to identify neurons, and comprehensively compared MDA and GenomePlex. Single neuronal nuclei or pooled 100-neuronal nuclei samples from three normal adult human brains (UMB1465, UMB4638, and UMB4643), one trisomy 18 (tri18) fetal brain (UMB866), and single lymphoblast cells (GM21781) were subjected to either MDA or GenomePlex, followed by low-coverage whole-genome sequencing (WGS) and CNV analysis (Table S1). Amplification uniformity was assessed by the distribution of copy-number (CN) ratios (*CNR_i_*) of 6,000 “equal-read” bins across the genome (Baslan et al., 2012). Compared to unamplified “bulk” DNA, one-cell and 100-cell samples subjected to MDA or GenomePlex showed significantly nosier CN profiles, suggesting that both methods introduce amplification noise (Figure 1; Figure S1). However, after GC normalization, the amplification noise did not show obvious bias to any particular genomic region at ~500 kb resolution (Figure S1D), with the exception of chromosome 19 by MDA, which shows systematically reduced CN (not shown). Amplification noise introduced by GenomePlex was noticeably lower than MDA, such that amplification of a single cell with GenomePlex produced noise similar in magnitude to pooled 100-cell samples amplified with MDA (Figures 1B and 1C; Figure S1) (Van der Aa et al., 2013).

### Measuring Amplification Quality with a Median Absolute Pairwise Difference Algorithm

In order to quantitate CN noise, we adapted a QC metric designed for microarray data, the median absolute pairwise difference (MAPD) algorithm (Affymetrix, 2008). MAPD measures the absolute difference between the log2 CN ratios of every pair of neighboring bins and then takes the median across all bins. Higher MAPD scores reflect greater noise, typically associated with poor-quality samples. MAPD provides significant advantages over other standard sample deviation measures such as SD, median absolute deviation, and interquartile range, since it is more robust to the presence of real CNVs.

As expected, unamplified bulk samples showed the best (lowest) MAPD scores (mean = 0.06 ± 0.02, n = 3), and the MAPD scores of GenomePlex-amplified one-cell samples (mean = 0.20 ± 0.05, n = 54) were consistently lower than MDA one-cell samples (mean = 0.45 ± 0.17, n = 89) (Figure 1B; see also Figure S1E), consistent with the tighter distribution of CN ratios previously observed with PicoPlex/GenomePlex (Figures 1A and 1B) (Voet et al., 2013). In addition to the higher MAPD scores seen in MDA samples, there was a tail of MDA samples with very high MAPD scores, suggesting poor or nonuniform amplification (Figure 1B; see also Figure S1E). In fact, all MDA samples were subjected to an initial multiplex-PCR sample quality control (QC) prior to sequencing analysis (Figure S1A), and all the samples that failed this initial QC also had high MAPD scores (not shown), suggesting that the MAPD reliably measures the quality of the amplified genome.

We tested different genomic bin sizes to optimize the ability of single-cell WGS to detect CNVs, using the MAPD as a guide. With decreasing bin sizes, including ~500 kb, ~150 kb, and ~60 kb (with comparable read counts per bin), MAPD scores increased for both methods, suggesting greater noise with smaller bin sizes (Figure 1C). Such change in CN noise is not due to Poisson error of sequencing depth, because the number of read counts in each bin at various bin sizes is comparable, and because MAPD scores from unamplified bulk samples do not change with bin size (Figure 1C). Although MDA always gave higher MAPD scores, this can be partially compensated by increasing bin size, at the cost of CNV resolution. MAPD scores of MDA samples with ~500 kb bins were similar to GenomePlex samples at ~60 kb bin size (0.33 ± 0.02 and 0.33 ± 0.01, respectively; error = ±SD) (Figure 1C). For CN profiling in MDA single-cell samples, ~500 kb bin sizes represent a compromise that should reliably detect megabase-sized CNVs, sufficient for chromosomal aneuploidy and large CNV studies, while analysis of smaller CNVs is limited to GenomePlex samples.

### Chromosomal Aneuploidy Analysis of Single Neurons

A total of 215 cells were analyzed, including 97 single neurons from three normal adults, 18 single cells from a tri18 fetus, 24 cultured single lymphoblast cells from a normal adult, and 46 neurons and 30 non-neuronal cells from a patient with HMG and a somatic chromosome 1q CNV (Table S1). Only samples with MAPD ≤ 0.45 were included in genome-wide CNV analyses (Figure 1B), which included all GenomePlex samples. MAPD scores of MDA samples varied between different individuals (Figure 1B; see also Figure S1F), suggesting that postmortem interval, tissue handling, and other factors may influence DNA integrity and amplification. This intersample variability highlights the importance of quality controls prior to single-cell analyses and suggests low-coverage single-cell CNV analysis as a method to assess the quality of postmortem tissues for single-cell studies.

**Figure 2:**
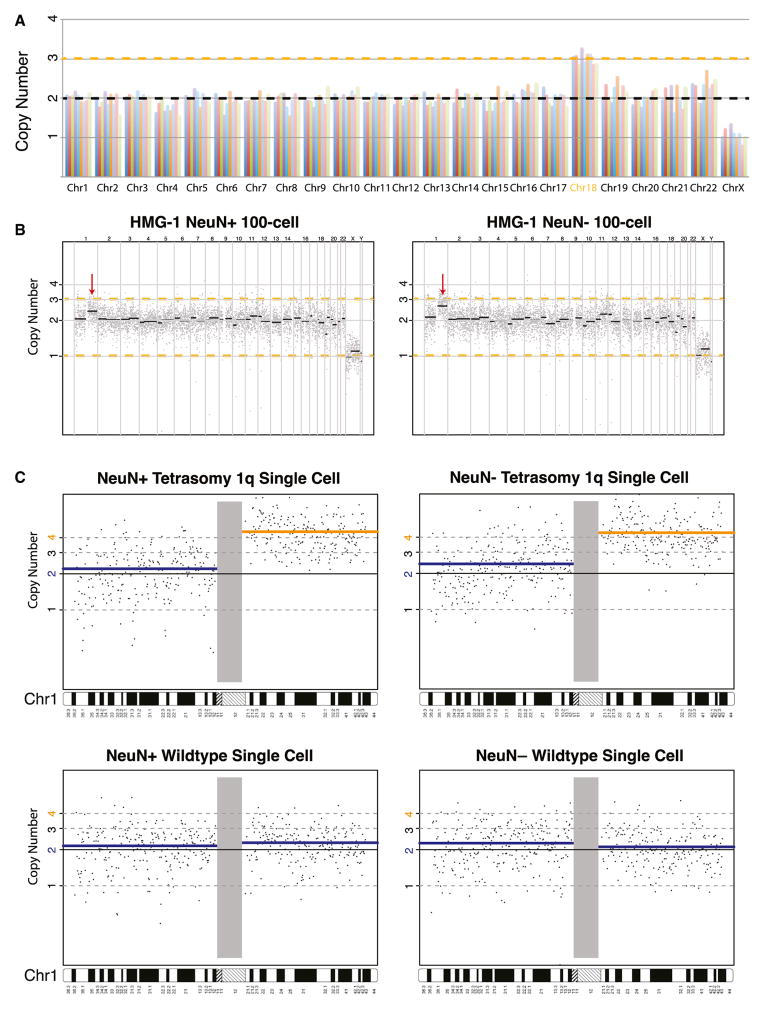
Chromosomal Aneuploidy and CNV Analysis of Single Neurons from Trisomy 18 and 1q CNV Brains (A) All nine single neuron genomes with germline trisomy 18 showed CN increase at chromosome 18. Chromosome CN of single cortical neurons from a tri18 (UMB866, 47XY, +18) individual demonstrates 100% sensitivity in detecting the CN gain at chromosome 18. CNs are normalized to the median CN of each chromosome across the five wild-type single neurons, with autosomes adjusted to a median CN of 2. Orange line denotes CN 3. (B) The 100-cell samples from HMG-1 brain, carrying a clonal 1q CNV, show that both NeuN+ and NeuN− populations showed noninteger CN increase at chr1q (red arrow). Gray dots denote CN of each genomic bin, and black lines denote the medium CN of each chromosome arm. Orange dashed lines denote CN 1 and 3. (C) CN plots at chromosome 1 of single-cell samples from both the NeuN+ and NeuN− populations showed four copies of chr1q in a fraction of samples and normal CN 2 in the rest of the samples. Blue and orange lines denote median CN of each chromosome arm. The blue line represents chromosome arms with CN 2, and the orange line represents chromosome arms with CN 4.

A total of 9 of 18 tri18 single neurons amplified by MDA passed the MAPD quality control, and all nine showed estimated CN of 3 at chromosome 18 (average CN of all tri18 single neurons = 3.01 ± 0.17 at chromosome 18) (Figure 2A; see also Figures S2A–S2C), with no strong evidence for aneuploidy of other chromosomes. The 100% sensitivity and specificity in calling tri18 shows the ability of single-cell WGS to detect large chromosomal CNVs after MDA. In contrast, of 82 single cortical neurons (from three normal individuals) with MAPD ≤ 0.45 amplified by either MDA or GenomePlex, 78 out of 82 were euploid (95.1%) (Figure 1B). No neurons showed discrete, integral CNV of an entire chromosome suggestive of simple trisomy or monosomy. Instead, three neurons showed genome-wide CN imbalances (Figures S2D–S2G) not limited to a single method or individual. Similar observations from other labs with both methods suggest that these cells are unlikely to represent simple aneuploidy, but their interpretation is uncertain (Baslan et al., 2012; McConnell et al., 2013). One neuron amplified by MDA showed an equivocal CN change at chromosome 19 (data not shown), but the systematic bias seen at chromosome 19 across MDA samples suggests that this might reflect a technical artifact.

### Analysis of a Large, Clonal, Pathogenic Somatic CNV

HMG is a brain overgrowth syndrome caused in some cases by brain-specific somatic SNVs or somatic CNVs of chromosome 1q (Lee et al., 2012; Poduri et al., 2012). Two cases previously studied by us showed a mosaic CN increase at chromosome 1q, originally interpreted as mosaic trisomy of 1q (Poduri et al., 2012). Neuronal and non-neuronal 100-cell MDA samples showed intermediate CN gain of the chromosome 1q arm in both populations (Figures 2B and 2C; see also Figures S2H and S2I), estimated at 2.35 and 2.7, respectively. This result confirmed the mosaic gain and suggested that the CNV arose in a cell giving rise to both neurons and non-neuronal cells, similar to our previous observation of a mosaic E17K point mutation in *AKT3* (Evrony et al., 2012).

CNV analysis of single nuclei from the brain of HMG-1, known to have been through multiple freeze-thaw cycles before analysis, was complicated by poor tissue preservation, reflected in the FACS scatterplot of HMG-1 (data not shown). Many HMG-1 cells amplified poorly and did not pass the MAPD QC threshold. Of 46 single neurons (NeuN+) sequenced, only 9 passed the threshold of MAPD of ≤ 0.45; one of these nine neurons was positive for the chromosome 1q CNV (Figure 2C), and eight showed normal CN for 1q (Table S1). Despite these limitations, CNV analysis showed clear *tetrasomy* of chromosome 1q (CN = 4) rather than the expected trisomy (CN = 3). Additional NeuN+ and NeuN− cells with higher MAPD scores confirmed the CN gain of 1q as a tetrasomy, rather than trisomy (Figure 2C). Tetrasomy of 1q was previously described in a mosaic state in humans with nasopharyngeal teratomas (Beverstock et al., 1999) as an isodicentric chromosome 1q [47, XX, +idic(1)(q10)]. Although FISH was not possible in this poorly preserved tissue, two different HMG samples (HMG-1 and HMG-2) show the exact same chromosome 1q gain with a centromeric breakpoint, suggesting they share the same isodicentric chromosome 1q tetrasomy. This surprising result highlights the power of single-cell analyses to define CN states of somatic mutations.

### Subchromosomal CNV Analysis of Single Normal Neurons

In order to optimize CNV calling, we performed CNV analysis at subchromosomal resolution in 24 single lymphoblasts and four ten-cell samples of cultured lymphoblasts derived from a normal male (GM21781). Unamplified DNA at the same passage was sequenced at the same read depth as amplified single-cell and ten-cell samples (average read depth of ~1.5 million unique reads per sample). A nonreference 2.15 Mb CN gain at distal chromosome Xp was identified in the bulk sample (Figure S3A) and validated in WGS data from the Personal Genome Project from this individual (https://my.pgp-hms.org/profile/huAE6220) (Ball et al., 2012). The same CN gain was called in four out of four ten-cell samples and 15 out of 24 one-cell samples, demonstrating reasonable sensitivity in detecting subchromosomal CNVs down to ~2 Mb resolution from single cells amplified by GenomePlex (Figure S3A). Additional high-confidence CNVs were shared by multiple single-cell samples but were absent or present at a low level from bulk and ten-cell samples, demonstrating sensitive detection of clonal, mosaic CNVs down to 1–2 Mb (Figure S3B).

**Figure 3:**
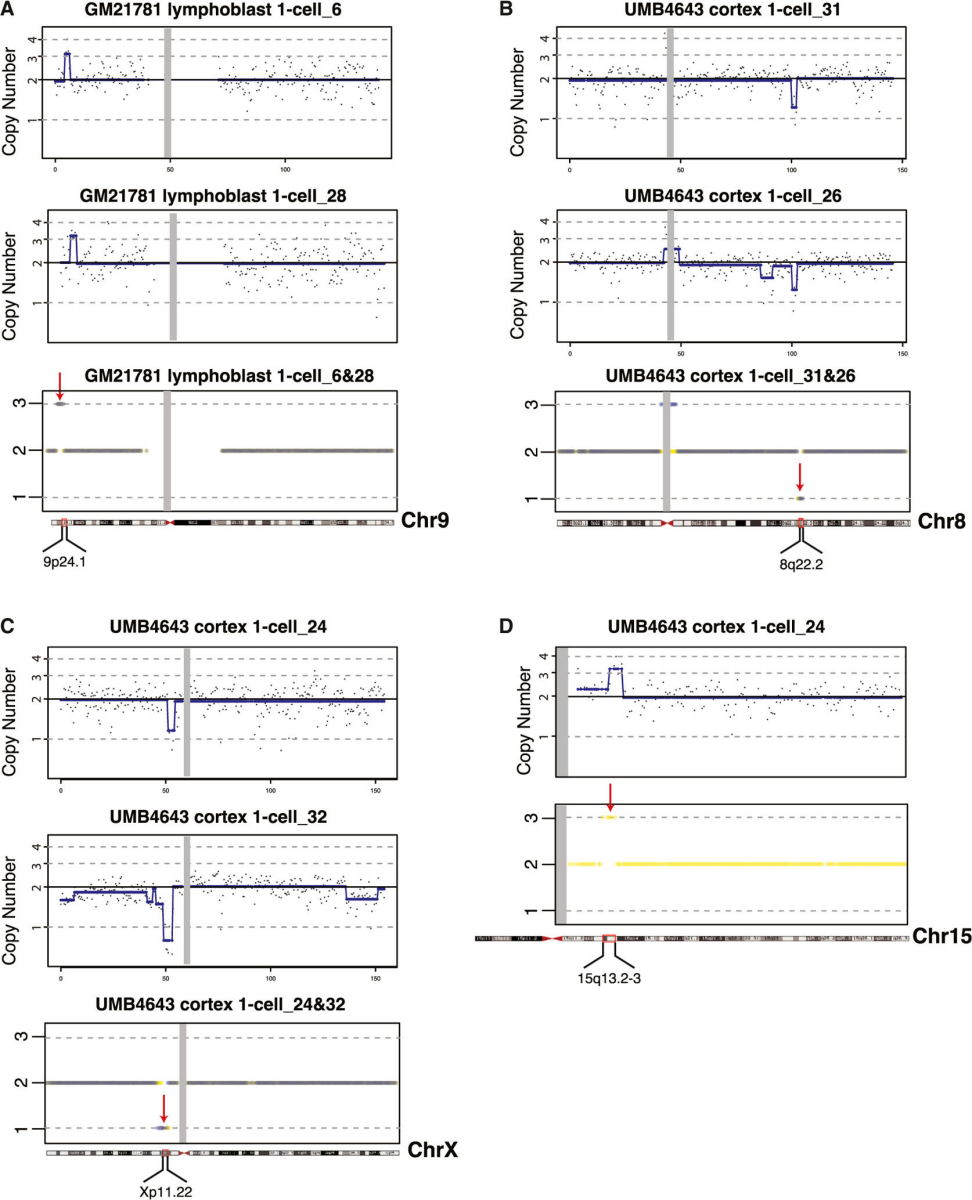
Mosaic Clonal CNVs Identified from Normal Lymphoblast Cells and Neurons (A) Example of a clonal CNV identified from lymphoblast GM21781. A ~1.9Mb CN gain at chr9 (chr9: 4,250,567–6,148,270) (indicated by red arrow) shared by multiple lymphoblasts (also see Figure S3B). Gray dots denote raw CN of each bin, and the blue line denotes the segmentation means of each CN segment, used for CN calls. The gray rectangle roughly defines boundaries of centromere. The bottom panel overlays CN calls of both single lymphoblasts. (B) Clonal CNV identified in UMB4643 cortical neurons. A ~1.9 Mb CN loss at chr8 (chr8: 100,204,912–102,089,812) (indicated by red arrow) is shared by two single neurons from 4643 cortex. The bottom panel overlays CN calls of both single neurons. A false-positive call at the centromere was filtered from the final call set. (C) Clonal CNV identified in UMB4643 cortical neurons. A ~2.3 Mb CN loss at chrX (chrX: 51,160,992–53,500,796) (indicated by red arrow) is shared by two single neurons from 4643 cortex. The bottom panel compares CN calls of both single neurons, emphasizing that they are closely in register. (D) 15q13 duplication identified in a single UMB4643 cortical neuron. An ~3 Mb CN gain at chr15 (chr15: 30,231,607–33,177,781) (indicated by red arrow), identified in a single neuron from UMB4643 cortex, overlaps the site of a recurrent CNV associated with ASD. The bottom panel shows the CN calls of UMB4643 cortex 1-cell_24 on chromosome 15.

**Figure 4:**
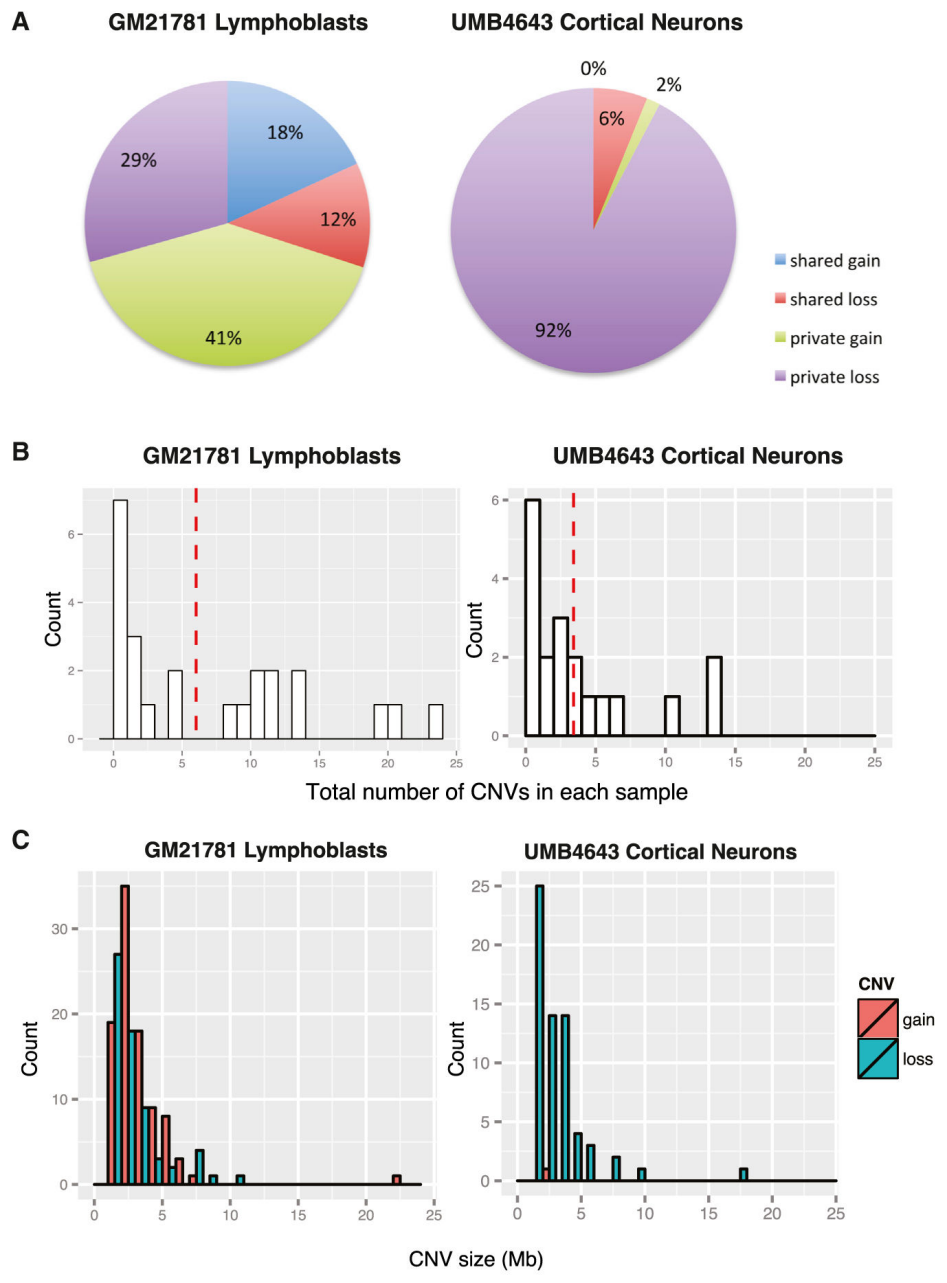
Comparative CNV Landscape in Lymphoblasts and Cortical Neurons (A) Pie chart summarizes CNVs identified from single lymphoblasts versus single neurons. Approximately 30% (18% CN gains and 12% losses) of CNVs identified from single lymphoblasts are clonal, whereas only 6% (6% CN losses and 0% gain) of CNVs identified from single neurons are clonal. CNVs identified from single neurons are predominately private losses, whereas CNVs identified from single lymphoblasts are balanced between losses and gains. (B) Histogram of total candidate CNVs identified in each single cell. Left: histogram of CNVs identified in each single lymphoblast (n = 24) shows that most single cells harbor less than five CNVs, though three outlier cells show larger numbers. The red dashed line shows the average CNVs per lymphoblast (6.7). Right: histogram of CNVs identified in each single neuron (n = 19), showing that most single neurons harbor less than five CNVs, with three outliers showing larger numbers. The red dashed line represents the average number of CNVs per neuron at 3.4. (C) Size distribution of CNVs identified from single cells. Left: size distribution of all CNVs identified in single lymphoblasts (n = 160). Most are less than 5 Mb with one outlier at 22 Mb, without obvious differences in size distribution between losses (green bars) and gains (red bars). Right: size distribution of CNVs identified in single neurons (n = 65). Most are <5 Mb with one outlier at 18 Mb. The only gain (red bar) identified is on the small end of the size distribution.

Among 24 single lymphoblasts, an average of 6.7 candidate CNVs were identified per sample, including 3.9 CN gains and 2.8 losses per sample, with ~30% of the candidate CNV events identifiable in more than one single-cell sample (Figure 3; see also Figure S3). A small fraction of samples showed many events (Figures 4A and 4B), but the number of CNVs identified in each single cell sample was not correlated with MAPD score (Figure S4). CNVs ranged from 1.3 Mb to 22.9 Mb, with a median of 2.4 Mb across all samples (Figure 4C). While we did not perform extensive validation of private candidate CNVs, the single-cell CNV analysis shows that germline CNVs, shared clonal CNVs, and private candidate CNVs as small as ~2 Mb can be identified in single-cell genomes with GenomePlex, confirming other reports that somatic CNVs are common in cultured cells (Abyzov et al., 2012).

The same CNV analysis performed on 19 euploid single cortical neurons amplified by GenomePlex showed an average of 3.4 candidate CNVs per single neuron, with 31% (6/19) of neurons lacking large CNVs (Figures 4A and 4B). Neuronal CNVs were predominately CN losses, with only 1 gain out of 65 candidate CNVs (Figures 4A and 4C). The size of candidate neuronal CNVs was similar to lymphoblasts, (median 2.3 Mb; range 1.7–17 Mb) (Figure 4B). Only two CNVs (6%) identified from single neurons were shared, each by two neurons (Figures 3B, 3C, and 4A). Nonetheless, sharing of CNVs by neurons validates them and suggests that large CNVs may arise in progenitor cells during normal brain development, consistent with the pathogenic CNV in HMG. Finally, we investigated whether any of the neuronal candidate CNVs correspond to known recurrent CNVs and identified a 2.9 Mb gain at 15q13.2-13.3 (Figure 3D), which overlaps a recurrent CNV associated with autism and other neuropsychiatric disorders (Sanders et al., 2011; Sebat et al., 2007).

## DISCUSSION

Our data confirm that somatic CNVs occur in the brain and that large-scale CNVs can be detected at a single-neuron level (McConnell et al., 2013) but extend these findings to show that CNVs can be shared by multiple neurons, both in pathological (HMG) and normal brain, showing that CNVs can be generated during neurogenesis and inherited clonally. Clonal CNV inheritance provides important validation for CNV calls, addresses the mechanism of CNV formation, and provides a mechanism whereby single CNV events could be shared by many neurons and hence be potentially deleterious.

Detection of CNVs in single neurons is inherently challenging, since all methods for analysis of single cells require amplification, so that the unperturbed state of the single neuronal genome cannot be queried. Several lines of evidence suggest that many of our candidate CNV calls are bona fide. First, single-cell WGS is highly sensitive and specific in detecting germline tri18 as well as the single Y chromosome in males, showing that the method detects large CNVs. Second, the method detects a clonal, somatic subchromosomal CNV of chromosome 1q associated with HMG that was independently discovered by microarray and qPCR analysis of unamplified brain tissue (Poduri et al., 2012). Third, GenomePlex (Baslan et al., 2012; Navin et al., 2011) can identify candidate CNVs in cultured lymphoblasts, including a germline CNV that was independently verified by analysis of unamplified DNA, and mosaic clonal CNVs that are shared by multiple single cells and technical controls. Finally, we identify candidate CNVs in single neurons and show that two of these are shared by two neurons each, providing independent validation of each CNV in a second cell and suggesting that some CNVs are generated during brain development.

Methodologies for single-cell genome amplification and analysis are rapidly evolving, and several different methods are available, such as MALBAC (Zong et al., 2012) and MIDAS (Gole et al., 2013). The MAPD score allows quantitative analysis of uniformity of amplification, adding a universal marker to compare different single-cell amplification methods to other standard metrics such as genome coverage, GC bias, and allele dropout. The MAPD metric quantitated how GenomePlex provided more even genome amplification, and more accurate CNV calls, as shown by others (Navin et al., 2011; Van der Aa et al., 2013). In our hands, MDA amplifies virtually the entire genome, but CNV calling requires relatively large bin sizing to overcome the nonuniformity of amplification and hence can only resolve large CNVs. Nonetheless, MDA detects tri18 accurately (average CN of nine cells = 3.01), suggesting that the nonuniformities of MDA are smoothed out over larger genomic regions and after computationally controlling for GC bias.

Although megabase-range somatic CNVs are seen in both cultured lymphoblasts and wild-type neurons, the CNV landscape differs between the two cell types. CNVs from lymphoblasts are more frequently clonal, whereas neuronal CNVs are more often private. This difference could be technical, but likely reflects cultured lymphoblasts’ continuous proliferation with ongoing generation of clonal CNVs, whereas the postmitotic nature of adult neurons only allows clonal CNVs to develop during development. On the other hand, we show that clonal CNVs can cause HMG, and clonal CNVs also occur occasionally in normal neurons, suggesting that some CNVs occur during development. Candidate neuronal CNVs in our data show a similar size range as lymphoblast CNVs but are biased toward losses compared to gains (McConnell et al., 2013). Although some candidate CNVs may reflect amplification dropout artifacts, the neuron-lymphoblast differences suggest that distinct molecular mechanisms regulate formation of CNVs in these two cell types.

Single-cell WGS showed that the 1q CNV in an HMG sample was likely a tetrasomy rather than a trisomy and hence present in fewer cells than initially suspected (~33% of non-neuronal cells and <20% of neurons), illustrating how a clonal somatic CNV in a minority of brain cells can cause widespread dysfunction (Poduri et al., 2012). Other brain malformations with intractable epilepsy are associated with SNVs in as few as 10% of cells assayed in blood or in brain (Gleeson et al., 2000; Jamuar et al., 2014; Kurek et al., 2012; Lee et al., 2012; Poduri et al., 2012, 2013). De novo CNVs are commonly associated with neuropsychiatric disease (Gilman et al., 2011; Levy et al., 2011; Sanders et al., 2011; Sullivan et al., 2012), and one candidate single-cell CNV gain (Figure 3D) involves proximal chromosome 15q, a region subject to recurrent CNV associated with neuropsychiatric disease. If such clonal CNVs occur early in development, they could involve enough cells to cause neuropsychiatric disease and yet be difficult to detect in blood.

## EXPERIMENTAL PROCEDURES

### Tissue Sources and Cell Isolation

Fresh-frozen postmortem tissue of three normal individuals (UMB1465, UMB4638, and UMB4643) and a tri18 fetus (UMB866) were obtained from the National Institute of Child Health and Human Development Brain and Tissue Bank for Developmental Disorders at the University of Maryland (Baltimore, MD). Fresh-frozen brain tissue of an HMG brain (HMG-1) was obtained in accordance with requirements of the institutional review boards of Boston Children’s Hospital and Beth Israel Deaconess Medical Center. An Epstein-Barr virus-transformed B-lymphoblast cell line GM21781, derived from a normal individual (PGP8, huAE6220) from the Personal Genome Project, was obtained from Coriell Cell Repositories and cultured with RPMI 1640 + 15% fetal bovine serum + 2 mM L-glutamine. Isolation of single nuclei from primary brain tissues and quality assessments were performed as described previously (Evrony et al., 2012). Single cells from B-lymphoblast suspension cultures were suspended in 1×PBS with 1×live-cell DNA dye DRAQ5, incubated at 37°C for 5 min per the manufacturer’s instruction (Cell Signaling Technology), then sorted into 96-well PCR plates preloaded with lysis buffer.

### scWGA by MDA and GenomePlex

All scWGA was carried out in a UV-treated laminar flow cabinet, and all surfaces, plastics, and nonbiologic buffers were UV treated for at least 30 min. MDA was performed as previously described (Evrony et al., 2012). GenomePlex-WGA4 was performed according to the manufacturer’s instructions (Sigma-Aldrich), with some modification to the lysis condition and reaction volume. Individual cells and pooled 10-cell and 100-cell controls were sorted into 96-well plates preloaded with 4 μl alkaline lysis buffer (100 mM KOH, 5 mM EDTA, 40 mM dithiothreitol), and neutralized with 1 μl neutralization buffer (400 mM HCl, 600 mM Tris [pH 7.5]). Fragmentation, adaptor ligation, and PCR amplification were performed according to manufacturer’s instruction with all reaction volumes reduced by half. Amplified products were purified with AMPureXP beads and quantified by nanodrop. Amplification quality and contamination were assessed by 1.5% agarose gel electrophoresis of 5% of the product. Samples with no visible product were excluded from library preparation. Four-locus multiplex PCR was performed on all MDA-amplified samples as described previously (Evrony et al., 2012). Samples with less than three loci amplified were generally excluded. Three single-neuron samples with one or two loci amplified were included in library preparation and sequencing to test the effectiveness of multiplex-PCR as a quality control.

### Whole-Genome Sequencing

WGS libraries prepared from 500 ng of DNA with the NEXTflex DNA sequencing kit (Bioo Scientific) were barcoded for multiplexed sequencing at the Harvard Biopolymers Facility (Harvard Medical School) and TUCF Genomics Center (Tufts Medical School) on HiSeq 2000 sequencers (Illumina). For low-coverage sequencing, 32 samples were multiplexed into each HiSeq lane for single-end 50 bp runs, obtaining ~5 million pass-filter reads per sample.

### CNV Analysis

The CNV analysis is adapted and modified from Baslan et al. (2012). Equal-read bins with variable sizes (6,000, 20,000, and 50,000) were created based on simulated reads from the hg19 reference genome (Baslan et al., 2012; Navin and Hicks, 2011) with median bin sizes of 458 kb, 137 kb, and 54 kb, respectively. Generating equal-read instead of “equal-size” genomic bins controls for read mappability so that each bin receives the same number of mappable positions, and hence similar read counts from each bin are expected from low-coverage WGS. The percentage GC content of each calculated bin (*GC_i_*, where *i* stands for individual bins) was computed for GC normalization.

All sequence reads were demultiplexed by CASAVA, allowing for one mismatch in the 6 bp index sequences. Sequence reads were mapped with bowtie with the following settings: *bowtie -v 2 -m 1–best–strata* (Langmead et al., 2009). PCR duplicates were removed using SAMTools (Li et al., 2009). Reads per bin (*RPB_i_*) for each sample were computed and the CN ratio (*CNR_i_*) for each bin was calculated as *CNR_i_ = RPB_i_/Median(RPB)*, where *i* stands for individual bins. *CNR_i_* was further normalized based on the GC content of each bin (*GC_i_*) to yield the GC-normalized CN ratios (*nCNR_i_*).

### MAPD Metrics

The MAPD QC metric was developed by adapting the Affymetrix *m*ultiple *a*bsolute *p*airwise *d*ifferences algorithm (Affymetrix, 2008): *MAPD* = *Median*(|*log2nCNR_i+1_* − *log2nCNR_i_*|), where *i* stands for individual bins. A MAPD threshold of 0.45 was used for all single-cell samples. All three MDA single-cell samples that failed the initial multiplex-PCR QC exhibited very high MAPD scores, confirming that multiplex-PCR can serve as a first-pass QC method that eliminates some, but not all, poor-quality samples.

### Chromosomal CN Calling

Chromosome CN ratios for each chromosome arm were calculated as the fraction of reads in each sample aligning to the chromosome arm, normalized to method-specific normalizer reference sets. Raw read counts for each chromosome and chromosome arm were obtained by SAMTools (Li et al., 2009) using hg19 centromere coordinates obtained from the UCSC genome database. For MDA samples, the median fraction of reads aligning to each chromosome arm across five euploid cortical neurons from normal individual UMB1465 was used as the normalizer reference set. For GenomePlex samples, four ten-cell samples from GM21781 lymphoblasts were used as the normalizer reference set. For autosomal chromosomes, chromosome CN ratios were multiplied by two to obtain the absolute CN of the diploid genome. Sex chromosome CNs were determined by their CN ratios to the autosomes within each sample. The final CN of each chromosome arm or chromosome were obtained by rounding to their nearest integer.

### CNV Calling

For CNV calls, the GC-normalized CN ratios (*nCNR_i_*) were log2 transformed and segmented using the circular-binary segmentation (CBS) algorithm provided in the DNAcopy package under the following settings: *alpha* = *0.02, nperm* = *1000, undo.splits* = *“sdundo,” undo.SD* = *1.0, min.width* = *5* (Baslan et al., 2012; Venkatraman and Olshen, 2007). CNVs were called based on the absolute distance between the segment mean of each genomic segment and the median segment mean across all segments of each sample: *Seg.dist_j_* = |*Seg.mean_j_* − *Median(Seg.mean)*|, where *j* stands for individual segments. A CNV was called if a given genomic segment met the following criteria: (1) *Seg.dist_j_* ≥ *2 MADs* (*m*edian *a*bsolute *d*istance) of the segment means of the sample; (2) p value of the segment mean being different from the median segment mean across all segments <0.1 based on one-tail Z-test: *pnorm(Seg.dist_j_/sd(Seg.mean))* ≥ *0.9*; (3) spans four or more genomic bins; (4) ≥1 Mb in size; and (5) does not overlap with centromere regions. All these parameters were empirically calibrated to obtain >60% detection of a 2.7 Mb germline CNV in GM21781 single lymphoblast samples (Figure S3). Further increase of CNV calling stringency resulted in reduced sensitivity, while further decreasing the calling stringency produced excessive private CNV calls with noninteger segment means, suggestive of false positives. Finally, the segment mean of called events was converted to diploid genome CN and rounded to the nearest integer to represent the final CN. For male samples, sex chromosomes were treated separately from autosomes. Clonal CNVs were identified by searching for CNV calls between different samples that share at least two genomic bins; the p value for such events to happen by chance is <10^−4^ based on an ANOVA multisample test.

## Supplementary Material

Supplementary Figure 1-4 and Table 1
