# Supplementary material for: Single-Cell, Genome-wide Sequencing Identifies Clonal Somatic Copy-Number Variation in the Human Brain
Source: Cell Rep. Author manuscript; Available in PMC 2014 Dec 19. (PMC4272008; doi:10.1016/j.celrep.2014.07.043)
Supplement: Supplementary Figure 1-4 and Table 1 [file NIHMS643222-supplement-Supplementary_Figure_1-4_and_Table_1.pdf]

Supplementary Figure 1 (Related to Figure 1)

A) Quality control workflow of single neurons amplified by either MDA or GenomePlex prior to chromosomal copy number analysis. 4-loci multiplex PCR is performed on all MDA amplified samples; samples in which < 3/4 loci amplified were considered of low quality and eliminated from further analysis. GenomePlex amplified samples with no yield were also eliminated from downstream analysis based on Nanodrop quantitation and agarose gel. Additional quality control based on the MAPD matrix was then applied to all single cell samples amplified by either method prior to chromosomal copy number analysis.

B) Distribution of copy number ratios of all genomic bins across the genome from bulk, 100-cell and 1-cell samples amplified by MDA, and 1-cell samples amplified by GenomePlex. Kernel density plot shows that all samples have their median copy number ratios approximately 1 as expected. Bulk sample shows the tightest distribution indicating the lowest copy number variability across bins; whereas the 1-cell MDA sample shows the broadest copy number ratio distribution indicating the highest copy number variability, among all sample types. 1-cell GenomePlex performs similarly to 100-cell MDA, consistent with observation in **Fig. 1**.

C, D) Pre and post-GC normalization of copy number profile on 1-cell MDA sample. GC normalization effectively corrects most of the regional bias introduced by MDA amplification with the exception of Chr19.

E) Histogram of MAPD score of unamplified bulk (red), GenomePlex amplified single neurons/cells (blue), and MDA amplified single neurons (green). MAPD scores closely mirror the copy number noise observed in **Fig. 1A**, with unamplified bulk being

least noisy and MDA single neuron samples being most noisy. Average MAPD score of unamplified bulk samples is 0.06 (n=3), of GenomePlex amplified single neurons/cells is 0.2 (n=54), and of MDA single neurons is 0.45 (n=89). A long tail, marked by an arrow, is observed in MDA single neurons, indicating poor quality samples. X-axis represents the MAPD score and y-axis represents the counts.

F) Histogram of MAPD scores of MDA amplified single neuron samples from different individuals, showing significant tissue-to-tissue variability after MDA amplification. n=18 for UMB866 cortex; n=7 for UMB 1465 cortex; n=32 for UMB 4638 cortex and n=32 for UMB4343 cortex. The red dash lines denote the mean MAPD score of each tissue sources.

Supplementary Figure 2 (Related to Figure 2)

A) Boxplot of chromosomal copy numbers of trisomy 18 single neurons (n=9) and wildtype single neurons from 3 individuals (n=79). 3 neurons with genome-wide aberrations were excluded from the plot and no additional chromosomal aneuploidy event was detected except for an equivocal copy number loss at chromosome 19 from one neuron (marked by red asterisks). The lower and upper hinges of the boxplot represent the 25th to 75th percentile (IQR) of chromosome copy numbers across all samples; lower and upper whiskers represent lower or upper hinges + 1.5*IQR.

B, C) Examples of copy number profiles at chromosome 18 from wildtype (UMB4643) and trisomy 18 (UMB866) single cortical neurons. Black dots denote raw copy number for each bin; blue and orange lines denote median copy number of each chromosome arm at expected copy number 2 and 3, respectively.

D, E, F, G) Whole genome copy number plots of the 3 aberrant neurons and one neuron with equivocal copy number loss at chr19. The black dash line indicates copy number 2 and orange dash lines indicate copy number 1 and 3.

H, I) Copy number plots at chromosome 1 of 100-cell sample from NeuN+ and NeuN- populations. Median copy numbers of chromosome 1q of NeuN+ and NeuN- samples are at 2.35 and 2.7, respectively.

Supplementary Figure 3 (Related to Figure3)

A) Examples of a 2.15Mb germline copy number gain being sensitively detected from 10-cell and 1-cell samples from GM21781 lymphoblasts.

B) Additional examples of a mosaic clonal copy number gain identified from GM21781 lymphoblast (related to Fig. 3A). A non-integer copy number increase is identified in some of the 10-cell samples.

Supplementary Figure 4 (Related to Figure 4)

Upper panel: Number of CNVs (in all types) detected per single lymphoblast genome is not correlated with the copy number profile noise measured by the MAPD matrix (R^2=0.05). X-axis represents the MAPD score of each single cell sample; and the Y-axis presents the number of CNVs identified of each single cell sample.

Lower panel: Number of CNVs (in all types) detected per single neuronal genome is not correlated with the copy number profile noise measured by the MAPD matrix (R^2=0.17).

Supplementary Table 1 (Related to Figure 1)

Summary of cells analyzed for this study. Over 200 cells from 6 different normal and diseased brains were studied. The proportion of cells that passed our QC filter (MAPD >0.45) and the proportion that were estimated to be grossly euploid are also shown.

Supplemental Figure 1.

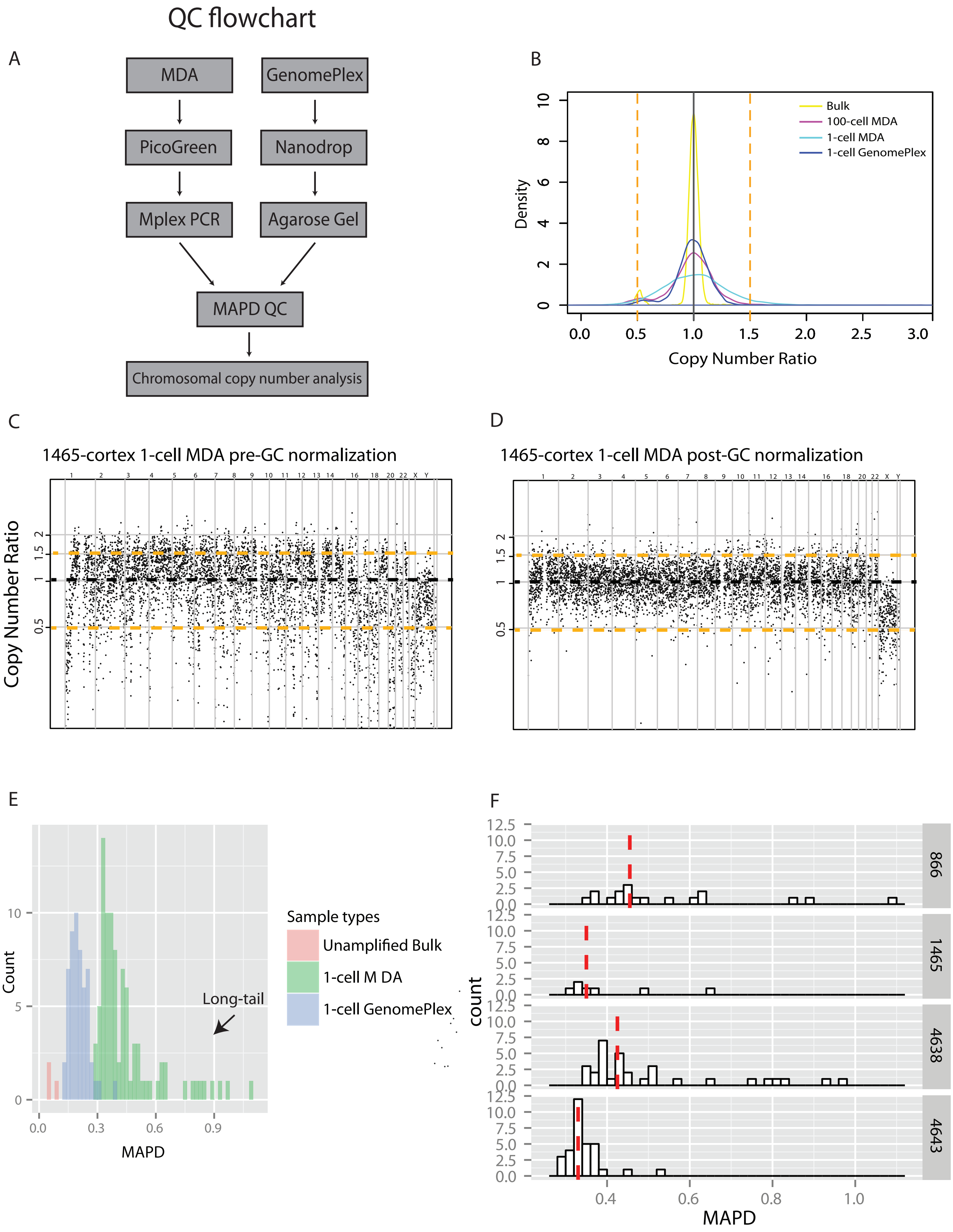

Supplemental Figure 2.

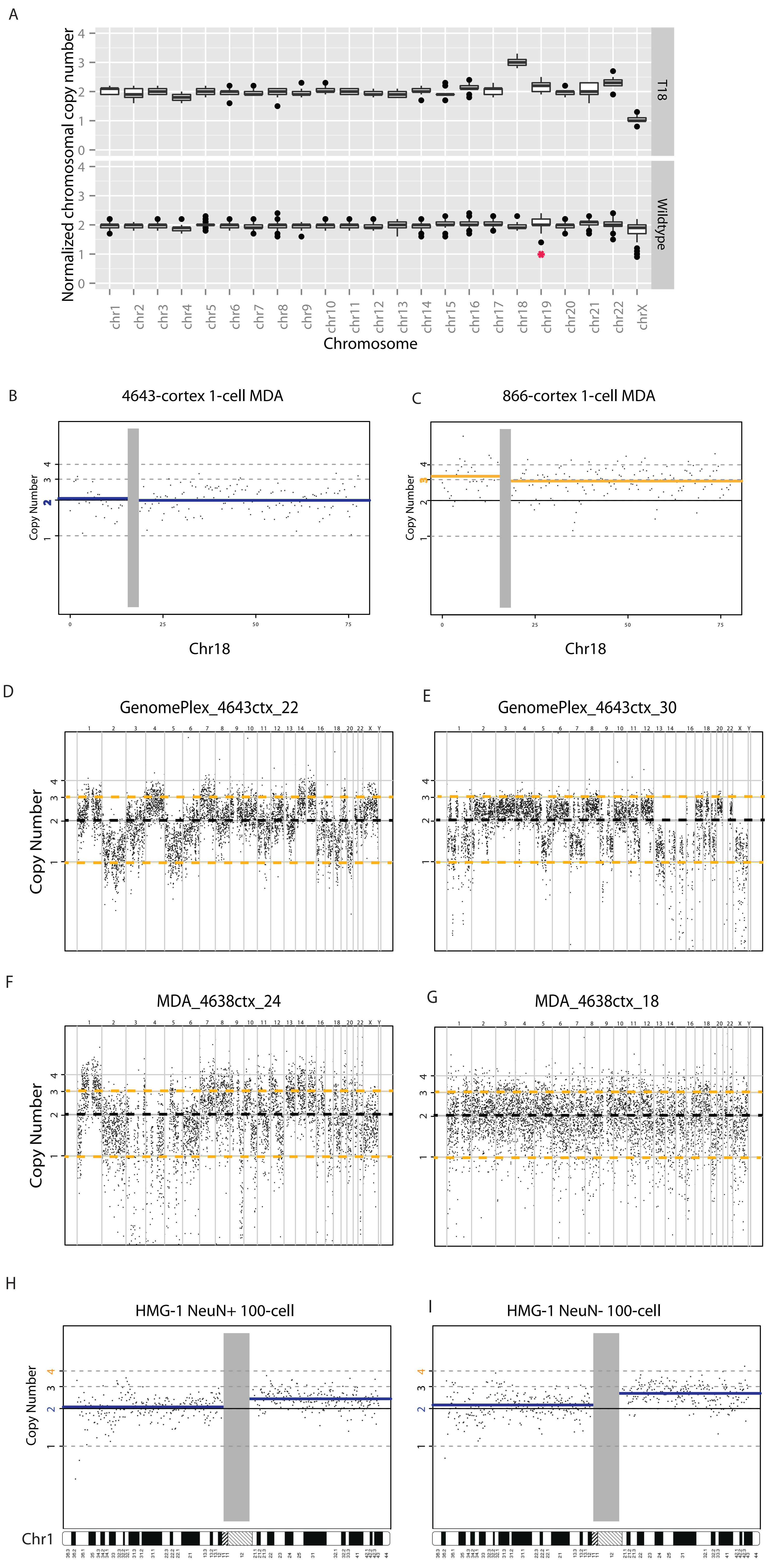

Supplemental Figure 3.

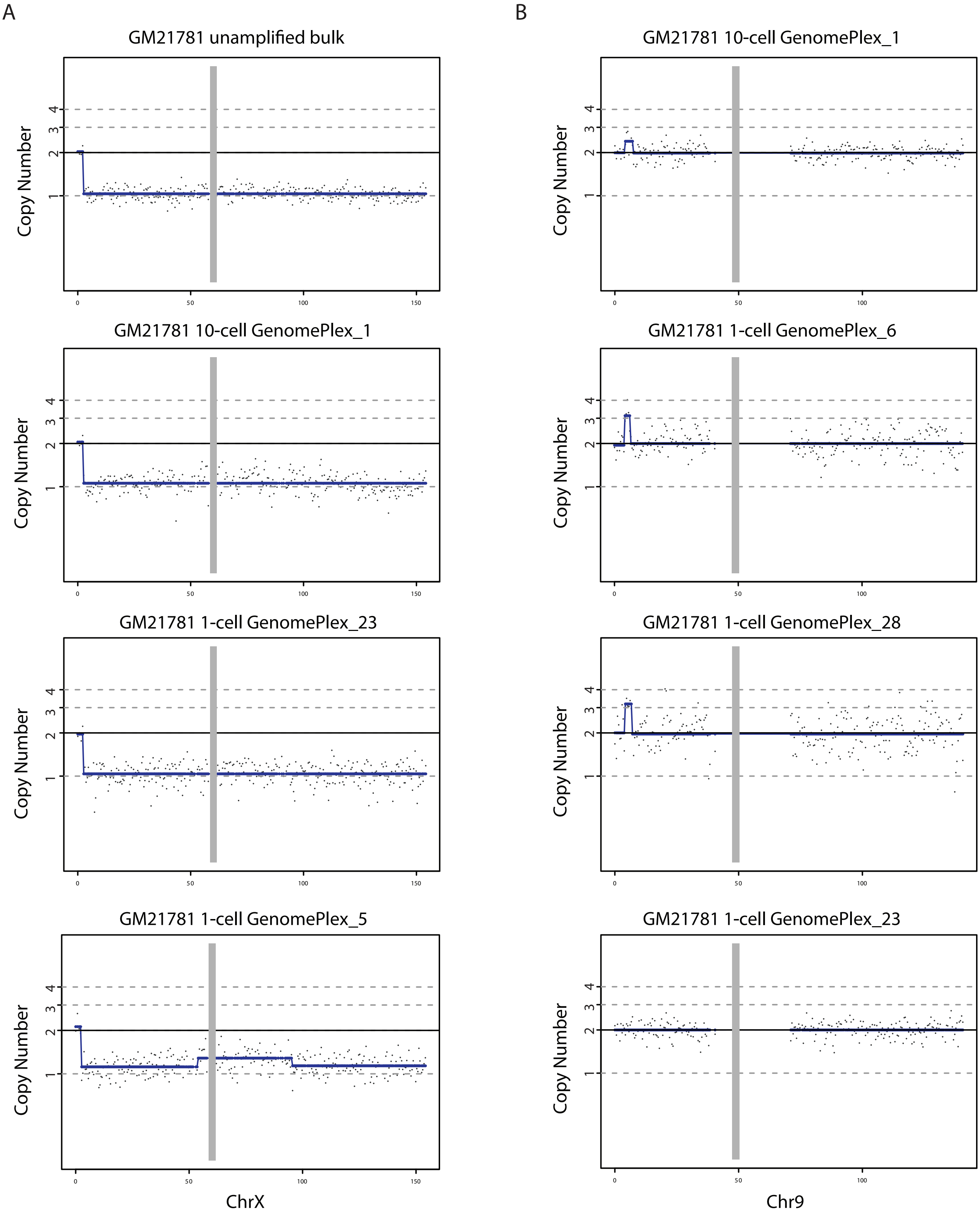

Supplemental Figure 4.

GM21781 Lymphocytes

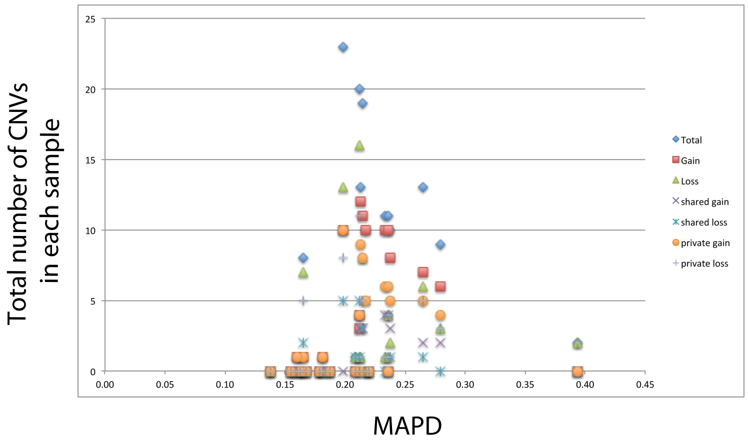

UMB4643 Cortical Neurons

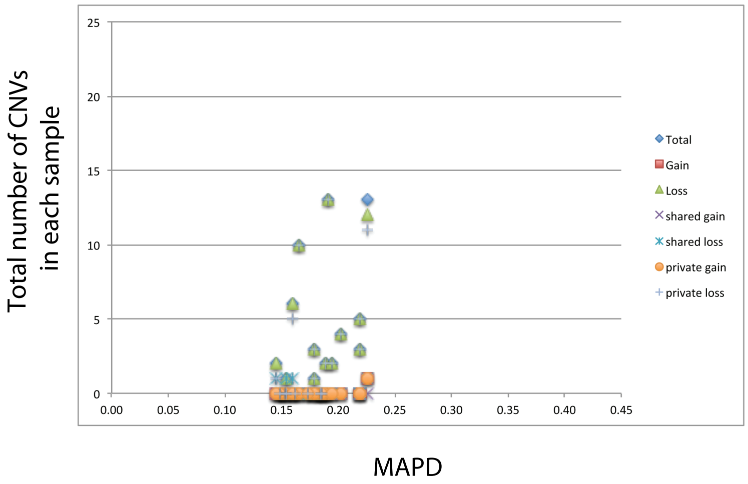

Supplemental Table 1. Summary of single cells analyzed for chromosomal copy numbers.

**Individual**	**Karyotype**	**Tissue type**	**Cell type**	**Amplification method**	**# of cells analyzed**	**# of cells passed QC (MAPD <= 0.45)**	**# of euploid cells**	**# of aneuploid cells**
**UMB1465**	46XY	Cortex	Neuron	MDA	7*	5	5	0
**UMB4638**	46XX	Cortex	Neuron	MDA	32	20	18	2
**UMB4643**	46XX	Cortex	Neuron	MDA	32	31	31	0
**UMB866**	47XY, 18	Cortex	Neuron	MDA	18	9	0	9**
**HMG-1**	46XY	Cortex	Neuron	MDA	46	9	8	1
**HMG-1**	46XY	Cortex	Glia	MDA	30	0	0	0
**UMB4643**	46XX	Cortex	Neuron	GenomePlex	26	26	24	2
**GM21781**	46XY	Cultured lymphocytes	B-lymphocyte	GenomePlex	24	24	24	0
				**TOTAL**	**215**	**124**	**110**	**14**

* 4 of these single cells were sequenced at >30X coverage

** trisomy 18
